# Sweet Melody or Jazz? Transcription Around DNA Double-Strand Breaks

**DOI:** 10.3389/fmolb.2021.655786

**Published:** 2021-04-20

**Authors:** Qilin Long, Zhichao Liu, Monika Gullerova

**Affiliations:** Sir William Dunn School of Pathology, University of Oxford, Oxford, United Kingdom

**Keywords:** double strand break, transcription, transcription factors, helicase, cohesin

## Abstract

Genomic integrity is continuously threatened by thousands of endogenous and exogenous damaging factors. To preserve genome stability, cells developed comprehensive DNA damage response (DDR) pathways that mediate the recognition of damaged DNA lesions, the activation of signaling cascades, and the execution of DNA repair. Transcription has been understood to pose a threat to genome stability in the presence of DNA breaks. Interestingly, accumulating evidence in recent years shows that the transient transcriptional activation at DNA double-strand break (DSB) sites is required for efficient repair, while the rest of the genome exhibits temporary transcription silencing. This genomic shut down is a result of multiple signaling cascades involved in the maintenance of DNA/RNA homeostasis, chromatin stability, and genome fidelity. The regulation of transcription of protein-coding genes and non-coding RNAs has been extensively studied; however, the exact regulatory mechanisms of transcription at DSBs remain enigmatic. These complex processes involve many players such as transcription-associated protein complexes, including kinases, transcription factors, chromatin remodeling complexes, and helicases. The damage-derived transcripts themselves also play an essential role in DDR regulation. In this review, we summarize the current findings on the regulation of transcription at DSBs and discussed the roles of various accessory proteins in these processes and consequently in DDR.

## Introduction

**FIGURE 1:**
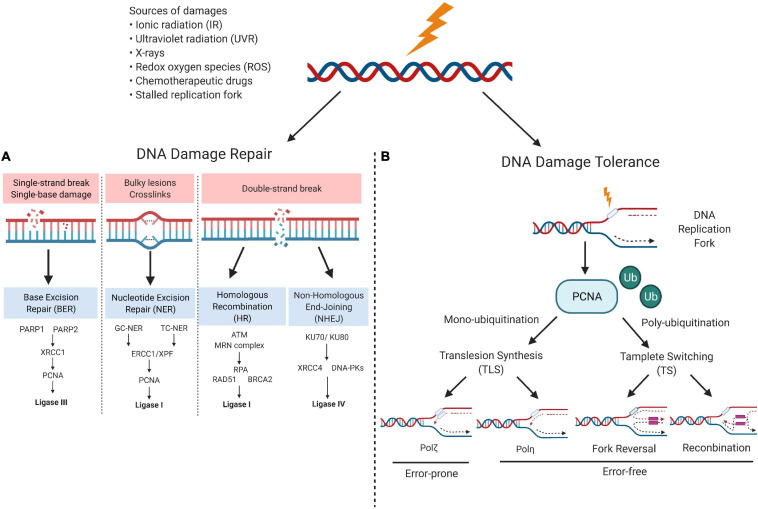
Sources of DNA damage and DNA damage response (DDR) network. **(A)** DNA damage repair choices. Small base alterations could be recognized and corrected through base excision repair (BER) pathway; longer base damages that disturb DNA-helix structure, such as bulky DNA adducts, are repaired through the nucleotide excision repair (NER) pathway; for double –strand breaks (DSBs) repair, homologous recombination (HR) and non-homologous end-joining (NHEJ) are two main repair mechanisms. **(B)** DNA tolerance pathway. Following persistent genotoxic stress, cells allow temporary DNA replication in the presence of unrepaired templates. Monoubiquitinated proliferating cell nuclear antigen (PCNA) facilitates translesion synthesis (TLS) while polyubiquitinated PCNA associates template switching (TS). Apart from Polζ-mediated TLS is an error-prone process, Polη-mediated TLS and TS are error-free processes (modified from reference Ghosal and Chen, 2013). Image created with BioRender.

Human genome integrity is constantly exposed to thousands of endogenous and exogenous molecular attacks, which could lead to DNA breaks. Human cells experience 70,000 DNA lesions per day, among which the majority are single-strand breaks (SSBs). Approximately 50 SSBs could also be converted into detrimental double-strand breaks (DSBs) during replication progress (Caldecott, 2008; Tubbs and Nussenzweig, 2017). DSBs are considered the most lethal lesions, leading to chromosome rearrangements and deletions and, consequently, to oncogenesis or cell death. The common sources of DNA damage include ionic radiation (IR), ultraviolet radiation, x-rays, redox oxygen species, chemotherapeutic drugs, and stalled replication fork (Ciccia and Elledge, 2010; Ketley and Gullerova, 2020). To counteract these pernicious cellular and external activities, DNA damage response (DDR) pathways developed to safeguard genomic fidelity by facilitating lesion site recognition, DNA damage repair, or DNA damage tolerance (DDT) (Ciccia and Elledge, 2010; Branzei and Psakhye, 2016) (Figure 1A).

In mammalian cells, small base alterations could be recognized and corrected through the base excision repair (BER) pathway, while bulky DNA adducts are repaired through the nucleotide excision repair (NER) pathway (Krokan and Bjoras, 2013; Scharer, 2013). Notably, SSBs are detected and repaired by a specialized BER signaling, termed as designated SSB repair (SSBR) (Giglia-Mari et al., 2011). DSBs can be repaired by homologous recombination (HR) or non-homologous end-joining (NHEJ). Specifically, HR mainly occurs at the S/G2 phase of the cell cycle and requires the existing sister chromatid as the template, which ensures the accuracy of the repair. On the other hand, in NHEJ, two broken DNA ends are joined together, in an error-prone process occurring throughout the cell cycle (Chapman et al., 2012). Unrepaired DNA lesions ultimately lead to stalling of replication forks, cell cycle arrest, and cell death. The DNA damage tolerance (DDT) pathway is a series of signals that enables the completion of DNA replication in the presence of unrepaired templates (Bi, 2015; Cipolla et al., 2016). In mammalian cells, at least two types of DDT evolved to overcome damage-induced replication blocks: one is translesion synthesis (TLS) and the other is template switching (TS). During the TLS process, the high-fidelity DNA polymerases are switched to specialized TLS polymerases with the ability to bypass the lesions. Admittedly, the TLS polymerases display intrinsic error-prone features, but the ultimate outcomes of TLS could be error-free or error-prone, depending on the ubiquitylation and SUMOylation status of proliferating cell nuclear antigen (PCNA). Contrary to the TLS, TS is a damage repair pathway that relies on the nascent DNA strand exchange between damaged and intact sister chromatid, leading to the reactivation of replication (Goodman and Woodgate, 2013; Bi, 2015; Cipolla et al., 2016) (Figure 1B).

The close relationship between the cell cycle and DDR has been well documented in recent years. When cells encounter severe DNA damage, cell cycle progression is restrained through activation of DNA damage checkpoints (Chao et al., 2017). Failure to activate or over-activation of cell-cycle checkpoints leads to tumorigenesis or cell death. The periodic activation and inactivation of cyclin-dependent Ser/Thr kinases (CDKs) is the metronome that controls the cell-cycle progression in both normal and damage conditions. The induced checkpoint activation correlates with cell cycle arrest, which allows cells to repair DNA (Shaltiel et al., 2015).

Recently, an increasing number of studies have suggested that RNAs could also play important roles in DDR. RNAs can be simply categorized into messenger RNAs (mRNAs) with encoding information for protein production and non-coding RNAs (ncRNAs). Depending on their length and origin, long ncRNAs (lncRNAs, >200 bp) and small ncRNAs (sncRNAs, <200 bp) can be further processed into microRNAs (miRNAs) and other types of RNAs exhibiting versatility in DDR (Ketley and Gullerova, 2020). miRNAs are reported to regulate gene expression by post-transcriptional gene silencing by targeting mRNAs of various key proteins involved in DDR (Van Kouwenhove et al., 2011). For example, miR-182 targets BRCA1, a core component in the HR pathway, and its overexpression could significantly delay repair kinetics, shift to NHEJ, and increase cell sensitivity to irradiation (Moskwa et al., 2011). Additionally, RNAs can modulate DDR pathways through a gene expression-independent manner (Tehrani et al., 2018). Drosha- and Dicer-dependent DSBs derived sncRNAs, termed as damage response RNAs (DDRNAs) (in mammalian cells) or diRNAs (in plants), facilitate the recruitment of DDR factors including MDC1, pATM, BRCA1, and 53BP1 to DSBs, further stimulating the DDR process at the mediator level (Francia et al., 2012; Wei et al., 2012). Furthermore, lncRNAs could interact with the complementary DNA strand to form DNA : RNA hybrid structures, which can have a positive effect on HR and/or NHEJ pathways (Ohle et al., 2016; Cohen et al., 2018; Lu et al., 2018; Yasuhara et al., 2018).

Initiation of transcription at the sites of DNA damage results in the production of specific DSB-derived RNAs that play an important role in DDR. Therefore, it is not surprising that transcription at DSBs is well controlled and regulated by multiple pathways (D’Alessandro and d’Adda di Fagagna, 2017; Machour and Ayoub, 2020). On the other hand, active transcription sites (ATS) possessing DNA structures, such as R-loops and G quadruplexes, are prone to generate DNA damage (De Magis et al., 2019; Miglietta et al., 2020). These structures are the sources for stalled replication forks, leading to endogenous DSBs (Gan et al., 2011; Liu et al., 2012). Notably, unpaired single-strand DNA strands lacking protection from exonucleases and endonucleases are often observed at ATS, causing an increased threat to global genome integrity (Lemmens et al., 2015). Therefore, transcription shut-off tends to occur when these structures are present (Cree and Kennedy, 2014; Skourti-Stathaki et al., 2019). In contrast, the accumulating evidence also shows that DNA:RNA hybrids and R-loops near the DSBs provide opportunities for the cells to maintain controlled DNA repair (Michelini et al., 2017; Burger et al., 2019). Remarkably, transcription itself was proven to directly affect DDR, ranging from the choice of repair mechanisms (HR or NHEJ) to the expression of damage-induced RNAs (Michelini et al., 2017; Yasuhara et al., 2018; Burger et al., 2019). Therefore, in this review, the focus is on the dynamic interaction between transcription and DSBs and how components of canonical transcriptional regulation could modulate the efficiency of DSB repair.

## Transcription Activity at DSBs

**FIGURE 2:**
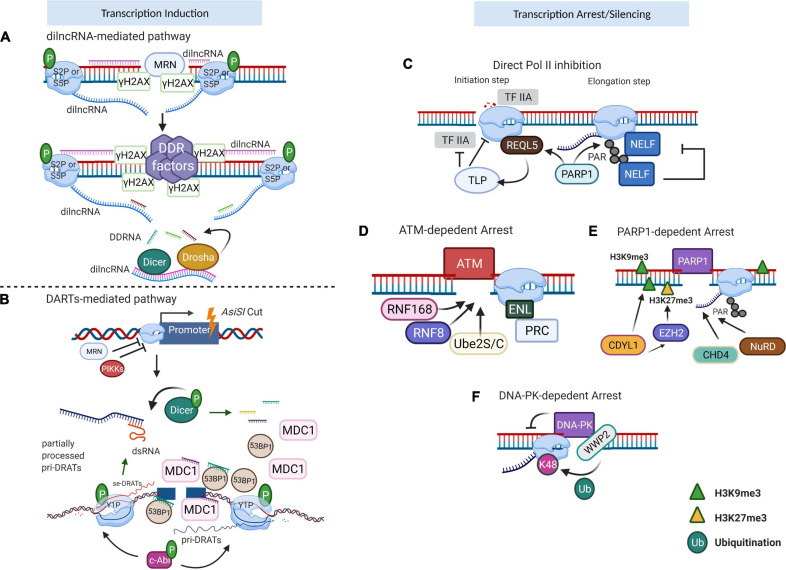
Transcription regulation near the DSBs. **(A)** dilncRNA-mediated transcriptional induction. When damage occurs, Mre-Rad50-Nbs1 (MRN) directs S2P/S5P to DSB, which conducts bidirectional generation of dilncRNAs. These dilncRNAs are further processed into damage response RNAs (DDRNAs) by Drosha and Dicer. DDRNAs complementary with pre-mature single-strand dilncRNAs, function as a recruiting signal for DDR factors. **(B)** DARTs-mediated transcriptional induction. DSBs produced by *AsiSI* enzyme stimulate phosphatidylinositol 3-kinase-related kinase (PIKK) signaling to inhibit the regular RNA polymerase II (RNAPII) transcription but activate c-Abl to facilitate Y1P CTD of RNAPII activity. Y1P generates pri-DARTs which hybrid with template DNA and correspondingly stimulates the production of se-DARTs. The free se-DARTs produced by p-Dicer cleavage of dsRNAs facilitate the recruitment of DDR factors such as MDC1 and 53BP1 to the DSB. **(C)** RNAPII inhibition after DNA damage. During the transcription initiation stage, REQL5 directly interacts with RNAPII at the DSB, acting as the recruiting signal for TLP which is a negative regulator of TFIIA. Once transcription progresses to the elongation stage, RNAPII PARylation is conducted by PARP1, which subsequently leads to NELF recruitment and shutdown of active transcription. **(D)** ATM-dependent transcription arrest. ATM triggers the accumulation of RNF8/RNF168/Ube2S/C, enforcing the pausing of actively transcribing RNAPII and consequently repressing the transcription activity nearby the breaks. **(E)** PARP1-dependent transcription arrest. CDYL1, recruited by PARP1, mediates transcription repression by deposition of H3K9me3. Also, CDYL1 promotes the accumulation of EZH2, further reinforced the transcription silencing with repressive H3K27me3. FRRUC advances the monoubiquitylation and facilitates the H2A.Z incorporation. **(F)** DNA–PK-dependent transcription arrest. DNA–PK could directly inhibit RNAPII bypass at I-*Ppo*I-induced DSB and impair RNA manufacture. WWP2, associated with DNA–PK, ubiquitylates RNAPII RPB1 at K48 to facilitate proteasome-dependent RNAPII eviction. Image created with BioRender.

Transcription at DSBs mediated by RNA polymerase II (RNAPII) results in the production of DSB-induced lncRNAs (dilncRNAs) (Figure 2A) or damage-responsive transcripts (DARTs) (Figure 2B) (Michelini et al., 2017; Burger et al., 2019; Pessina et al., 2019). As in the case of protein-coding genes, the activity of RNAPII is regulated by the modifications of its C-terminal domain (CTD) of the RNAPII largest subunit that consists of multiple heptapeptide repeats (Tyr1–Ser2–Pro3–Thr4–Ser5–Pro6–Ser7). The CTD plays roles in regulating the transcription elongation and termination and is extensively phosphorylated during the transcription cycle. Michelini et al. (2017) showed the presence of phosphorylated Serine 2 (S2P) or 5 (S5P) CTD of RNAPII at DSBs leading to the production of dilncRNAs in both directions, from the break and to the break, resulting in double-stranded RNA (dsRNA). Such dsRNAs can be further processed by Dicer and Drosha into DDRNAs. Interestingly, they proposed that single-stranded DDRNAs can in turn pair with single-stranded dilncRNAs. In their model, dilncRNAs serve as both DDRNA precursor and binding platforms (Francia et al., 2016; Michelini et al., 2017). The dilncRNAs increase the efficiency of HR repair by contributing to the generation of DNA:RNA hybrids. However, upon EXO1 and CtIP knock-downs, the hybrid formation was diminished, suggesting that the hybrid structures are formed by downstream of end resection (Li et al., 2016; D’Alessandro et al., 2018). Similar results were also observed after antisense oligonucleotide treatment targeting dilncRNAs. These data showed that dilncRNAs can hybridize with resected DNA ends and participate in the HR pathway after end resection. However, the ability of dilncRNAs to recruit downstream HR factors, such as RAD51, was not confirmed (D’Alessandro et al., 2018).

Another study showed that c-Abl kinase triggers the phosphorylation of CTD of RNAPII at Tyrosine 1 (Y1P) at DSBs (Burger et al., 2019). Y1P RNAPII transcribes lncRNAs in the direction away from the break. These lncRNA transcripts form R-loops close to their termination sites, which in turn function as the promoters for the transcription of the opposite DNA strand, leading to the formation of double-strand RNAs (dsRNAs) or damage-responsive transcripts (DARTs). The generation of dsRNA at DSBs can be visualized with the application of an anti-dsRNA-specific J2 antibody (White et al., 2014; Burger et al., 2017). Furthermore, the overexpression of RNase H, which specifically digests DNA : RNA hybrids, caused impaired dsRNA synthesis, demonstrating that R-loops trigger *de novo* transcription and are required for the dsRNA formation (Burger et al., 2019). Immunoprecipitation of mammalian nascent elongating transcripts (mNET-IP) associated with Y1P CTD of RNAPII confirmed that DARTs were suppressed after the treatment of cells with c-Abl inhibitor, suggesting that Y1P CTD is essential for DARTs synthesis. DARTs contribute to DDR through the recruitment of repair factors, such as p53-binding protein 1 (53BP1), and the mediator of DNA damage checkpoint 1 (MDC1) to the breaks (Burger et al., 2019). A more recent study showed that induction of DSBs results in the recruitment of complete RNAPII pre-initiation complex (PIC), MED1, and CDK9 to form functional promoters at DSBs. The absence or inactivation of these factors caused a reduction in DDR foci, both *in vivo* and *in vitro*. The dilncRNAs further recruited DDR proteins such as 53BP1 to the foci that showed liquid–liquid phase separation condensate properties. The assembly of DSB-induced transcriptional promoters stimulates RNA synthesis, which consequently leads to phase separation of DDR factors in the shape of foci (Pessina et al., 2019).

## Global Transcriptional Silencing Upon DNA Damage and Transcriptional Arrest Around DSBs

Ionizing radiation generates random DNA damage including nucleic acid alterations, SSBs, and DSBs (Ma et al., 2012). Another widely used approach to induce random DSBs is achieved using chemotherapeutic drugs, such as etoposide. Etoposide, topoisomerase II inhibitor, preferentially causes damage to CTCF-binding sites nearby or within the active transcriptional domain (Canela et al., 2017, 2019), affecting cells mainly in the S-phase during DNA replication (Thanasopoulou et al., 2012). Another approach is to use the controllable enzyme-mediated DSB induction at known genomic locations. Since the first application of I-*Sce*I mediated DSB published in 1994 (Rouet et al., 1994), various sequence-specific strategies to generate DSB at transgenic loci have been developed (Shanbhag et al., 2010; Gunn and Stark, 2012; Gelot et al., 2016). For example, a cell line that harbors one or two I-*Sce*I sites contributed to the discovery of the protective function of cohesin in preventing the distal end joining in the S/G2 phase (Gelot et al., 2016). Another cell line, harboring multiple tetracycline-response elements (TRE) sites together with the I-*Sce*I site and the MS2 site, was applied to monitor the transcripts and accessory proteins in both *de novo* active transcription and DSB-mediated transcriptional repression (Ui et al., 2015). Recently, Vitor et al. (2019) generated two reporter cassettes with I-*Sce*I and MS2/PP7 sequence to visualize individual transcripts on single-cell level at two single distinct locations: a promotor-proximal region and an exon region. Interestingly, they observed global transcriptional repression and *de novo* transcription at DSBs that was dependent on their genomic location: the transcription at promotor-proximal DSB was suppressed while DSB in the exon resulted in bidirectional transcription initiation (Vitor et al., 2019).

The *Asi*SI-inducible system was developed to investigate DSBs induced at selected endogenous loci rather than the transgenic positions (Iacovoni et al., 2010). This U2OS-based cell possesses a stable integrated *Asi*SI enzyme, which transfers from cytoplasm to nucleus and specifically cuts –GCGATCGC– sequences upon addition of 4-hydroxytamoxifen (4-OHT). BLESS and BLISS techniques confirmed the induction of 100∼200 canonical DSBs across the genome (Iannelli et al., 2017; Clouaire et al., 2018), allowing the study of transcriptional dynamics at selected DSBs (Aymard et al., 2014; Burger et al., 2019), site-specific end resection progress (Zhou et al., 2014), repair pathway preference (Aymard et al., 2014), and chromatin translocations (Cohen et al., 2018). Another site-specific restriction enzyme system is based on I-*Ppo*I endonuclease, generating 15 breaks at defined foci (Berkovich et al., 2007). With the application of I-*Ppo*I system, the distribution pattern of Nbs1 and phosphate-ATM has been revealed, and a DNA–PK-dependent transcriptional arrest was detected around the DSB (Berkovich et al., 2007; Pankotai et al., 2012; Caron et al., 2019a).

Since the DSBs can occur in both transcribed or transcriptional inactive regions in the genome, the retention of genome integrity relies on precise coordination between transcription and repair mechanisms. In general, the global repression signals block transcription initiation and/or elongation and lead to RNAPII termination (Shanbhag et al., 2010; Williams et al., 2015). Direct repression of transcription initiation or elongation is mediated by TATA-box binding protein-like protein 1 (TLP) (Suzuki et al., 2019), RECQL5 helicase (Aygun et al., 2008; Saponaro et al., 2014), or negative transcription elongation factor (NELF) complex (Williams et al., 2015) (Figure 2C). Specifically, the normal function of TLP is essential to suppress Pol II initiation, but the precise underlying signaling remains elusive (Suzuki et al., 2019). Early studies proved that RECQL5 can deactivate both transcription initiation and elongation (Aygun et al., 2008; Saponaro et al., 2014). Intriguingly, after laser damage, RECQL5 was also involved in DSB-proximal transcriptional arrest in a PARP1-dependent manner (Popuri et al., 2012; Khadka et al., 2015).

The transcriptional state around DSBs is tightly coordinated by multiple signals. The ataxia-telangiectasia mutated (ATM) kinase, poly (ADP-ribose) polymerase 1 (PARP1), and DNA-dependent protein kinase (DNA-PK) are critical components to mediate transcriptional arrests around the breaks (reviewed in reference Caron et al., 2019b). The pivotal step of ATM-dependent transcriptional arrest (Figure 2D) is the regulated accumulation of RNF8/RNF168/Ube2S/C, which can further recruit other chromatin-binding repair factors (such as 53BP1). This enforces the pausing of actively transcribing RNAPII and consequently represses the transcriptional activity nearby the breaks (Gatti et al., 2012; Mattiroli et al., 2012; Paul and Wang, 2017). Although the ATM kinase can also evoke the transcription repression at DSB sites in an RNF8/RNF168/Ube2S/C-independent manner, the understanding of its precise mechanism requires further analyses. BAF180, a subunit of chromatin-remodeling complexes of the SWI/SNF family, PBAF, together with polycomb-repressive complex (PRC), leads to transcriptional repression at the sites of DNA damage (Kakarougkas et al., 2014). The recruitment of PRC to the DNA breaks is regulated through the binding of phosphorylated ENL/AF9 to E3 ubiquitin ligase BMI1 and Ring1B (Ui et al., 2015). Chromodomain Y-like protein (CDYL1) is rapidly recruited to the damage sites in a PARP1-dependent manner (Figure 2E), mediating transcription repression by deposition of histone H3 trimethylated on lysine 9 (H3K9me3). CDYL1 also promotes the presence of enhancer of zeste homolog 2 (EZH2) at the breaks, leading to the accumulation of repressive histone methyl H3K27me3 to further reinforce the transcription silencing (Abu-Zhayia et al., 2018). The depletion of CDYL1 resulted in persistent G2/M arrest (Abu-Zhayia et al., 2018). The FBXL10-RNF68-RNF2 ubiquitin ligase complex (FRRUC) can repress transcription by monoubiquitylating H2A at Lys119 in non-damaged cells. FRRUC has also been reported to advance the monoubiquitylation and facilitate the H2A.Z incorporation in PARP1- and TIMELESS-dependent manner at the sites of DNA damage (Rona et al., 2018). These two processes are critical for transcription repression and HR-directed repair. Moreover, PARP1 recruits repressive chromatin modifiers, such as CHD4 (Polo et al., 2010) and NuRD (nucleosome remodeling and histone deacetylation complex) to DSBs (Chou et al., 2010; Gong et al., 2017). ZMYND8-NuRD only participates in HR repair (Chou et al., 2010). KDM5A-dependent H3K4me3 demethylation is a prerequisite for ZMYND8-NuRD binding to the damaged sites, and the lack of KDM5A leads to impaired HR (Gong et al., 2017). Contrary to the factors uniquely implicated in HR or NHEJ, the deletion of PBAF or NELF-E has been shown to affect both HR and NHEJ repair pathways (Kakarougkas et al., 2014; Awwad et al., 2017). Certain factors have a dual role in both transcription silencing and DNA repair. The aforementioned NELF complex is a typical example. PARP1 recruits NELF to the RNAPII complex, leading to transcription elongation pause and DNA repair through BRCA1 (Awwad et al., 2017; Bishara et al., 2021). Another factor is BAF180, which mediates ATM-dependent transcriptional silencing through the heterochromatin formation (Kakarougkas et al., 2014). Together with PBAF, cohesin is also required for modulating transcription near the DSBs in both G1 and G2 phases, while facilitating HR by holding the sister chromatids in close proximity (Meisenberg et al., 2019). DNA-PK also mediates the transcriptional arrest by inhibiting the bypass and processivity of RNAPII at I-*Ppo*I-induced DSBs, which subsequently impair translation through a proteasome-like manner (Pankotai et al., 2012) (Figure 2F). Recently, WWP2-dependent ubiquitylation of RPB1 has been reported as the signal for downstream RNAPII termination (Caron et al., 2019a).

Although multiple cellular signals facilitate transient downregulation of transcription in the proximity of that DSB, the damage-induced non-canonical local transcription can be initiated. More recently, accumulating evidence suggests that R-loops (consists of a DNA:RNA hybrid and a non-templated DNA strand) formed as transcriptional intermediates participating in the diverse cellular process, including mutagenesis, replication fork collapse, transcription termination, and preservation of genome integrity in both yeast and mammalian cells (Ginno et al., 2013; Wahba et al., 2013; Skourti-Stathaki et al., 2014). R-loops participate in checkpoint-mediated termination (Marabitti et al., 2020) and the pausing of RNAPII (Ohle et al., 2016; Awwad et al., 2017), further contributing to transcriptional silencing and DNA repair at the actively transcribed regions (Yasuhara et al., 2018). Despite the paused RNAPII might cause prolonged existence of R-loops near the break (Cohen et al., 2018), the *de novo* transcription from 3′ of resected end leads to the production of dilncRNA, which works as precursors for DDRNA (Michelini et al., 2017; D’Alessandro et al., 2018). Together with DNA repair proteins, the RNA species such as lncRNAs and DNA:RNA hybrids generated from the break also have a role in safeguard the faithful transcription (Chakraborty et al., 2016; Michelini et al., 2017; Lu et al., 2018). Notably, the pre-existing transcription status and the original position are accounts for diverse R-loop generation (Bader and Bushell, 2020). Therefore, the balanced relationship and interaction between transcription and repair are vital for genomic integrity.

## The Roles of Transcription Itself and Transcription Factors at DSBs

Despite the global transcription repression, the local transcription activation and transcription factors play critical roles in DDR. Treatment of U2OS cells with IR in the presence of 5,6-dichloro-1-b-D-ribofuranosylbenzimidazole (DRB) (a drug that inhibits RNAPII elongation) led to reduced Rad51 and RPA levels and impaired HR efficiency (D’Alessandro et al., 2018; Yasuhara et al., 2018). Similarly, treatment of cells with actinomycin D and triptolide led to a significant reduction of single-strand DNA (ssDNA) in the inducible *AsiSI-ER* endonuclease system (Aymard et al., 2014). Both studies showed a positive effect of transcription itself in repairing DNA lesions. Similarly, α-amanitin treatment leading to RNAPII degradation impaired the recruitment of early DDR factors such as 53BP1, XRCC4, and RAD52, and the biogenesis of DDRNAs (Francia et al., 2012; Chakraborty et al., 2016). Mirin, a Mre-Rad50-Nbs1 (MRN) inhibitor, reduced the amount of RNAPII at the damage sites, suggesting the function of MRN complex in RNAPII recruitment to DSBs (Michelini et al., 2017). Meanwhile, transcription itself was shown to facilitate the DNA end resection and promote the HR pathway *via* exosomes (Domingo-Prim et al., 2019). Domingo-Prim et al. (2019) showed that, in EXOSC10-depleted cells, hyper-stimulation of DNA end resection together with diminished RPA recruitment can be restored by transcription inhibitors and RNaseH1 overexpression respectively, suggesting that exosome-related RNA clearance is the precondition for regular RPA function, controlled end resection, and assembled HR (Domingo-Prim et al., 2019). Similarly, transcription inhibition can impair classical NHEJ (c-NHEJ). c-NHEJ factors were found to be recruited to transcribed regions and were preferentially associated with nascent RNAs (Chakraborty et al., 2016). *In vitro* experiments showed that RNAs hybridized to complementary DNA could act as a template and could bridge two broken DNA ends together to facilitate their repair. The absence of core c-NHEJ proteins, such as ligase IV, impaired the efficiency of RNA-templated DSB repair (Chakraborty et al., 2016). According to these results, c-NHEJ factors are preferentially recruited to transcribed regions, using RNA as a template to complete DSB repair. However, the transcription activation itself can compromise genome stability through the formation of R-loops or topoisomerase-induced DSBs.

General transcriptional factors (TFs) are involved in transcription regulation upon DNA damage (Table 1). For example, the DNA broken ends can function as promoters to recruits PIC, which recruits TFs (Pessina et al., 2019). Once an MRN complex recognizes DSBs, TBP (a component of TF II D which belongs to PIC) is first to be recruited to the promoter region during transcription initiation, which leads to the recruitment of RNAPII. The knockdown of general TFs, the inhibition of the MRN complex, or the treatment with transcription inhibitors, contribute to dilncRNAs reduction along with the reduced expression of 53BP1 (Pessina et al., 2019). Collectively, TFs facilitate nascent transcription by working as downstream targets of the MRN complex and participate in DDR through the promoter assembly at broken ends. CDK7, the catalytic subunit of TFIIH, modulates the phosphorylation of CTD of RNAPII at DSBs (Ebmeier et al., 2017). The CDK7 inhibition also leads to decreased transcription of HR factors, including RAD51, BRCA1, and BRCA2 (Shan et al., 2020). C-Myc, another TF, is believed to regulate more than 15% of total genes, some of which are DDR factors (Zeller et al., 2003; Perna et al., 2012; Chakravorty et al., 2017). Upon DNA damage, TET2 [a DNA dioxygenase that converts 5-methylcytosine (5mC) into 5-hydroxymethylcytosine (5hmC)] promotes DNA demethylation and interacts with C-Myc through the SIND1 bridge (Chen et al., 2018). The depletion of TET2 inhibits transcription of c-Myc-targeted DDR factors, such as BRCA1, making cells vulnerable to Cisplatin-induced DSBs, suggesting that the TET2-SIND1-c-Myc axis contributes to DDR at the transcriptional level (Chen et al., 2018).

**TABLE 1:** Transcription factors in DDR and their potential targets.

Transcription Factor	Targets	References
**GATA3**	CtIP	Zhang et al., 2017
**AR**	XRCC4, ATR, Rad51C, PARP1	Polkinghorn et al., 2013
**P53**	CDKN1A, GADD45A, BAX, PUMA	Sullivan et al., 2018
**E2F**	Rad51, CDK2, BRCA1, BARD1	Bracken et al., 2004
**TFEB/TFE3**	Rad9A, MDM2, BBC3	Jeong et al., 2018
**RUNX1/3**	GADD45A, CDKN1A	Samarakkody et al., 2020
**NF-kB**	HOTAIR	Ozes et al., 2016
**CTCF**	TERRA	Beishline et al., 2017
**Nrf-2**	53BP1	Kim et al., 2012
**Spl**	ATM, Nbs1	Beishline et al., 2012

Large protein complexes have also been reported to play distinct roles in transcription regulation upon DNA damage. The NELF, a four-subunit complex consisting of NELF-A, NELF-B, NELF-C/NELF-D, and NELF-E (Narita et al., 2003), mediates PARP1-dependent transcription arrest at DSBs (Awwad et al., 2017). Specifically, both NELF-E and NELF-A are rapidly recruited to DSBs, but only NELF-E interacts with PARP1 to repress the active transcription *via* the poly(ADP-ribosyl)ation (PARylation) at I-*Sce*I-mediated DSB presents an upstream of ATS. One striking feature is that the existence of RNAPII is required for the recruitment of NELF-E (Awwad et al., 2017). The 55 kD large isoform of CDK9 (CDK9 55K) was found to associate with Ku70 and its depletion caused the accumulation DSBs (Liu et al., 2010).

## Roles of Kinases and Helicases in Transcription Regulation at DSBs

Various kinases facilitate stable transcription during DDR through both direct or indirect control of other repair factors. In canonical DDR, the repair pathway starts from the recognition of exposed DNA ends by sensor complex MRN, which is followed by ATM, Ataxia-telangiectasia and Rad3-related (ATR), and DNA-PK initiation of various downstream DDR pathways (Blackford and Jackson, 2017). The kinase-associated transcription regulators largely rely on the activity of a particular kinase and become upregulated upon DNA damage. C-Abl, a kinase that phosphorylates RNAPII at Tyrosine 1 residue in non-damage conditions, is recruited to DSB sites and colocalizes with Y1P CTD RNAPII and γH2AX after 10Gy IR treatment. Depletion of c-Abl leads to decreased levels of Y1P at DSBs and consequently to the inhibition of DARTs generation. The impaired DNA damage repair could be rescued by c-Abl overexpression, demonstrating that c-Abl facilitates Y1P phosphorylation and DARTs transcription around DSBs (Burger et al., 2019). Besides, c-Abl was also reported to regulate p21 transcription in a p53-dependent manner in DDR, leading to cell cycle arrest and cellular senescence (Udden et al., 2014). Furthermore, PARP1, a crucial factor in DDR, is also targeted by c-Abl, leading to the induction of inflammatory genes transcription (Bohio et al., 2019).

The ATM kinase, another kinase with multiple roles in DDR, was found to phosphorylate various factors and regulates a wide range of biological processes, including transcription. The inhibition of ATM after DNA damage induction counteracts RNAPII stalling, promotes the accumulation of hyperphosphorylated RNAPII and decondensation of chromatin (Shanbhag et al., 2010). ATM represses transcription *in cis via* histone H2A monoubiquitylation or Lys63-linked polyubiquitylation (mediated by RNF8 and RNF168). The knockdown of these E3 ligases has been reported to partially reverse transcriptional silencing, confirming their role in transcription repression in DDR (Shanbhag et al., 2010). The RNF168 ubiquitinates H2A/H2AX at Lys13 and Lys15, but there is no direct evidence that polyubiquitylation at these two residues directly affects transcriptional silencing (Mattiroli et al., 2012). Upon DNA damage, ATM, together with MDC1 and NBS1, also mediates transcriptional repression of ribosomal-DNA (rDNA) through the defective Pol I initiation (Kruhlak et al., 2007). Another study also showed ATM-TCOF1-NBS1 signaling in response to CRISPR/Cas9-mediated rDNA breaks, repressing rRNA transcription and relocating rDNA into nucleolar caps (Larsen et al., 2014; Korsholm et al., 2019). When DSBs persist in transcriptionally active genomic regions, ATM mediates rDNA silencing, recruits the HR machinery throughout the cell cycle, and drives large-scale nucleolar reorganization (Harding et al., 2015; van Sluis and McStay, 2015).

DNA-PK, a key member of the phosphatidylinositol 3-kinase-related kinase (PIKK) family, is well known for its role in repair pathway choice between HR, NHEJ, and V(D)J recombination. Unlike ATM, DNA-PK specifically localizes nearby DSBs rather than being spread along chromatids (Weterings and Chen, 2007; Bjorkman et al., 2015). Transcription of genes containing site-specific DSBs generated by I-*Ppo*I endonuclease was rescued by the inhibition of DNA-PK, while the transcription of adjacent regions was not affected. This suggests that DNA-PK drives transcriptional arrest by inhibiting the bypass and processivity of RNAPII at DSBs in protein-coding genes (Pankotai et al., 2012). A recent study found that the RBP1 subunit of RNAPII is targeted by WWP2 for K48-linked ubiquitylation in response to DSB, which directs the proteasome toward RNAPII and consequently its termination (Caron et al., 2019a).

DNA helicases can regulate transcription termination and RNAPII pausing (Hatchi et al., 2015; Cristini et al., 2018). Specifically, senataxin was found to resolve DNA:RNA hybrids and prevent R-loops triggered DSBs. The knockdown of senataxin led to the accumulation of R-loops at both transcription termination regions and damage sites, suggesting that this helicase could counteract R-loop formation (Hatchi et al., 2015). Interestingly, the overexpression of RNaseH, which cleaves DNA : RNA hybrids, inhibits the recruitment of senataxin to those genomic loci, indicating the recruitment of senataxin to DSBs is R-loop-dependent (Hatchi et al., 2015). Moreover, depletion of DHX9 and XRN2 leads to a similar phenotype, confirming their roles in DDR-mediated R-loop formation (Skourti-Stathaki et al., 2011; Cristini et al., 2018). Additionally, senataxin depletion also inhibits the recruitment of HR factors, such as Rad51, to DSBs, stimulates the presence of NHEJ factors, and consequently modulates the repair pathway choice (Cohen et al., 2018).

DHX9, an SF2 type of the DExD/H-box family of helicases, dissociates complexes of DNA or RNA and heterogeneous polynucleotide structures in an ATP-dependent manner and has pivotal roles in repair pathway choice (Chakraborty and Hiom, 2019; Matsui et al., 2020). At the damage foci, DHX9 binds with BRCA1 to form the BRCA1-D complex, which recognizes nascent RNA. Given that the DHX9-mediated BRCA1 and RNAPII interaction also stimulates the initiation of DNA end resection, the end resection may be caused by paused or impaired transcription of the damaged region (Chakraborty and Hiom, 2019). Moreover, a recent study found that nuclear speckles protein, USP42, facilitates HR through BRCA1 loading and DNA end resection and interacts with DHX9 to resolve the break-induced R-loops, which highlights the importance of both DNA helicases and nuclear speckles in the modulation of the damage-associated transcriptional regulation (Matsui et al., 2020).

## Cohesin Recruitment to DSBs and Its Role in Transcriptional Regulation

Cohesin, one of the structural maintenance of chromosome (SMC) complexes, has been widely reported to be involved in eukaryotic chromatin processes. The recruitment of cohesin complex to DSBs can occur throughout the cell cycle, including the interphase, indicating that cohesin complex might play a distinct role in DDR besides its canonical function in establishing sister chromatin cohesion (Dorsett and Strom, 2012). Contrary to the widespread binding of yeast cohesin (over 50 kb), the human cohesin enrichment is restricted to a 5-kb vicinity surrounding the damaged lesions (Strom et al., 2004; Caron et al., 2012). The cohesin recruitment to DNA breaks was reported before, but the detailed recruitment mechanism remains elusive (Kim J.S. et al., 2002; Bauerschmidt et al., 2010). The diverse observations of cohesin-facilitating signaling around DSBs were observed in different species (Kim J.S. et al., 2002; Kim S.T. et al., 2002). Human cohesin complex subunit SA2, rather than SA1, is recruited to laser-induced damage sites in an Mre11/Rad50-dependent manner throughout interphase, while both cohesin subunits participate in intra-S checkpoint activation. SA2 binding to DNA is essential for HR, and its deletion increased the incidence of NHEJ (Kong et al., 2014). In contrast, in yeast, Smc1 is phosphorylated at serine 957 and 966 in an ATM-dependent manner, which also requires the presence of Nbs1 and Brca1 for the activation of S-phase cell cycle checkpoint (Kim S.T. et al., 2002).

**FIGURE 3:**
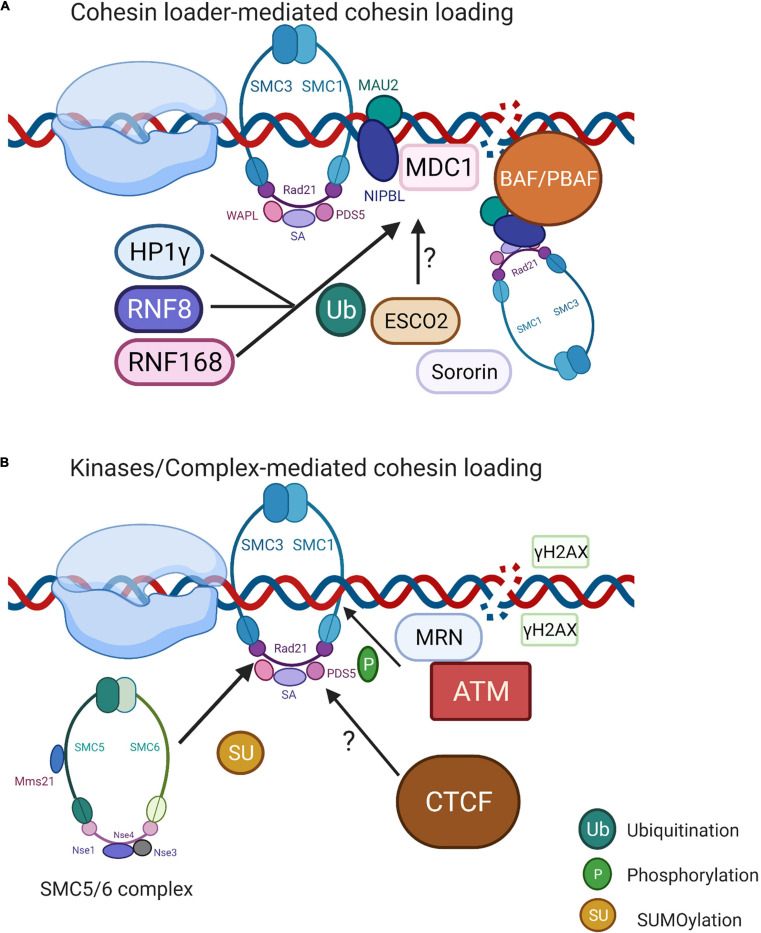
Localized cohesin recruitment at the DSBs. **(A)** NIBL-MAU2-mediated cohesin loading to DSBs. Cohesin loader NIBPL-MAU2 is reported to be recruited to DSBs through the MDC1-RNF168/RNF8-HP1γ complex. Another possibility is that RSC ortholog BAF/PBAF co-occupies the genomic locations of NIBPL-MAU2 and acts as the prerequisite for its loading. Whether ESCO2 and sororin also have functions in cohesin recruitment still requires further investigation. **(B)** Kinase/Complex-mediated cohesin loading. MRN complex activates ATM first, then recruits cohesin to DSB by phosphorylating SMC1 subunit. SMC5/6 complex (Mms21 subunit) regulates cohesin loading through SUMOylating cohesin subunit RAD21. CTCF is a rationale for cohesin recruitment toward chromosomal sites in non-damage conditions; however, its role in cohesin recruitment toward DSBs remains unknown. Image created with BioRender.

The heterodimer NIBPL-MAU2 (Scc2–Scc4 in yeast) is well known for its role in cohesin loading onto chromatin (Strom et al., 2004; Oka et al., 2011) (Figure 3A). The presence of NIBPL-MAU2 at DSBs is MDC1-, RNF168-, and HP1γ-dependent in human cells (Oka et al., 2011). RNF8 and RNF168 ubiquitylation are crucial for HP1γ-dependent NIPBL recruitment, as well as the ATM- or ATR-dependent phosphorylation of C-terminal HEAT domain of cohesin, which further stimulates its accumulation at the damage sites (Bot et al., 2017). In budding yeast, the chromatin remodeler RSC (remodels the structure of chromatin) colocalizes with the Scc2-Scc4 on chromatin, which further stimulates cohesin recruitment to DSBs (Oum et al., 2011). The orthologs of RSC in human are BAF and PBAF complexes, the critical members of SWI/SNF chromatin remodeling family, but whether these could directly recruit cohesin to the damage sites remains unclear. The mutation in NIBPL is a major cause of Cornelia de Lange syndrome (CdLS). Interestingly, CdLS shares clinical features with the Coffin–Siris syndrome, which is caused by the mutations in human SWI/SNF complexes. This could reflect the underlying interaction between cohesin loaders and RSC orthologs (Santen et al., 2012; Munoz et al., 2020).

The MRN complex, which recognizes DSB ends, activates ATM signaling and DNA repair, and shows the ability to stimulate cohesin recruitment to DNA. The subcomplex Mre11-Rad50 of the MRN complex is required for the recruitment of cohesin to DSBs by phosphorylating SMC1 subunit in human (Kim J.S. et al., 2002). A similar signaling pathway was observed in yeast, suggesting that the IR-induced phosphorylation of smc1 on Ser 957 and 966 residues was facilitated by ATM (Kim S.T. et al., 2002) (Figure 3B). The SMC1/3 phosphorylation mediated by ATM or ATR kinases further reinforced the binding of the cohesin complex to the genome (Kim et al., 2010). Intriguingly, the Mms21 subunit from SMC5/6 complex SUMOylates various lysine residues of cohesin subunit SCC1 (RAD21) recruited to DSBs, suggesting that SMC5/6 might be also involved in direct cohesin binding to DSBs (Wu et al., 2012). The search for specific regulators for yeast sister chromatid recombination after the endogenous induction of DSBs showed that both Rpd3L and Hda1 histone deacetylases (HDAC) participate in the DNA repair through cohesin loading and sister chromatid cohesion. The loss of Rpd3L directly affected the cohesin levels on chromatin, suggesting a general cohesin-loading mechanism by SCR regardless of DNA damage (Ortega et al., 2019). The chromatin modifications have an impact on cohesin association with chromatin, as heterochromatin promotes cohesin association with DNA in both yeast and higher eukaryotes (Bernard et al., 2001; Nonaka et al., 2002; Yi et al., 2018). Another versatile protein CTCF initially characterized as a transcriptional insulator, functions in transcriptional gene regulation, genome folding, RNAPII pausing, and imprinting. Twelve core nucleotides of CTCF share the consensus sequence with conserved cohesin DNA-binding sites (Parelho et al., 2008). In normal conditions, CTCF functions as a NIBL-MAU2-independent cohesin loader (Parelho et al., 2008; Rubio et al., 2008). At *Asi*SI-induced DSBs, CTCF contributes to reduced γH2AX spreading by creating a physical barrier. Cohesin binds to promoters of actively transcribed genes, limiting γH2AX establishment and stimulating transcription. The depletion of cohesin leads to an increased presence of γH2AX at promoters and impairs transcription (Caron et al., 2012). Collectively, cohesin interacts with CTCF upon DNA damage to modulate the formation of γH2AX and to regulate transcription.

Deletion of RAD21 or SMC3, the core subunits of the cohesin complex, resulted in an increased transcription nearby *Fok*I-induced DSBs (Meisenberg et al., 2019). A similar phenotype is observed after the knockdown of PDS5B and SA2, leading to reduced ubiquitinoylation of H2AK119, which can also be detected in PBAF-deficient cells (Meisenberg et al., 2019). Depletion of cohesin and PBAF can trigger chromosome rearrangements, especially when DSBs are localized at transcriptionally active regions. These results suggest that both cohesin and PBAF contribute to transcription silencing during DDR, indicating a functional correlation between cohesin and chromatin remodeling factors (Kakarougkas et al., 2014; Meisenberg et al., 2019). Additionally, the repair of rDNA lesions required the transcriptional repression *via* cohesin or human silencing hub (HUSH) complex-dependent signaling. The depletion of cohesin or HUSH complex led to a reduction in nucleolar caps and rRNA levels, which then affected the end resection in S/G2 cells (Marnef et al., 2019).

## Concluding Remarks and Future Perspectives

Double-strand breaks (DSBs) initiate complex coordination between recognition of the breaks, DDR network, signaling cascades of transcription, and its regulatory factors. Because of the diversity of chromatin content and structure and the complexity of RNAPII CTD, transcription at DSBs has to be tightly regulated, whether it is a burst of localized non-canonical transcriptional activity at DSBs or global canonical transcriptional repression across the genome. This undoubtedly implicates numerous cell-signaling factors, including transcription factors, kinases, DNA helicases, and chromatin-remodeling complexes. Furthermore, numerous studies indicated that the cohesin complex plays an indelible role in DDR, well beyond canonical sister chromatid cohesion in HR. More recently, accumulating evidence indicates that RNAs function in DNA damage repair; thus, it is essential to study not only the function of individual RNA but also the transcription of these precursors. Modified RNAPII is required for the generation of long non-coding transcripts at DSBs, which are subsequently processed into DDRNAs to mediate DDR (Francia et al., 2012; Wei et al., 2012; Francia et al., 2016). The dilncRNAs have been found to interact with its template DNA to form DNA : RNA hybrid to promote HR (D’Alessandro et al., 2018). Therefore, future studies are essential for further understanding regulatory mechanisms that control transcription and production of various RNA species at DSBs. As inefficient DNA repair can lead to oncogenesis, further understanding of molecular mechanisms of DDR is likely to provide the stepping stones for future cancer therapy.

## Author Contributions

ZL and QL wrote the initial draft of the manuscript. MG edited the manuscript. All authors contributed to the article and approved the submitted version.

## Conflict of Interest

The authors declare that the research was conducted in the absence of any commercial or financial relationships that could be construed as a potential conflict of interest.

## Abbreviation List

lncRNA, long non-coding RNA; DARTs, damage-responsive transcripts; dilncRNA, DSB-induced lncRNA; diRNA, damage-induced RNA; dsRNA, double strand RNA; mRNA, messenger RNA; miRNA, microRNA; ncRNA, non-coding RNA; sncRNA, small non-coding RNA; lncRNA, long non-coding RNA; DDRNA, damage response RNA; rDNA, ribosomal-DNA; ssDNA, single-strand DNA; RNAPII, RNA polymerase II; S2P, RNA Pol II phosphorylated at Serine 2; S5P, RNA Pol II phosphorylated at Serine 5; Y1P, RNA Pol II phosphorylated at Tyrosine 1; DDR, DNA damage response; DSB, double-strand break; SSB, single-strand break; BER, base excision repair; SSBR, single-strand break repair; HR, homologous recombination; NHEJ, non-homologous end joining; DDT, DNA damage tolerance; TLS, translesion synthesis; TS, template switching; ATM, ataxia-telangiectasia mutated; ATR, ataxia-telangiectasia and Rad3-related; ATS, active transcription site; CDK, cyclin-dependent Ser/Thr kinase; CDYL1, chromodomain Y-like protein; c-NHEJ, classical NHEJ; DNA-PK, DNA-dependent protein kinase; DRB, 5,6-dichloro-1-b-D-ribofuranosylbenzimidazole; EZH2, zeste homolog 2; FRRUC, FBXL10-RNF68-RNF2 ubiquitin ligase complex; H3K9me3, histone H3 trimethylated on lysine 9; HDAC, Hda1 histone deacetylases; IR, ionic radiation; MDC1, mediator of DNA damage checkpoint 1; mNET-IP, immunoprecipitation of mammalian nascent elongating transcripts; MRN complex, MRE11–RAD50–NBS1 complex; NELF, negative transcription elongation factor; NuRD, nucleosome remodeling and histone deacetylation complex; PARP1, poly (ADP-ribose) polymerase 1; PARylation, poly(ADP-ribosyl)ation; PCNA, proliferating cell nuclear antigen; PIC, preinitiation complex; PIKK, phosphatidylinositol 3-kinase-related kinase; PRC, polycomb repressive complex; RSC, remodels the structure of chromatin; SMC, structural maintenance of chromosome; TF, transcriptional factors; TLP, TATA-box binding protein-like protein 1; TRE, tetracycline response elements; 53BP1, p53-binding protein 1.
